# Point Prevalence of Pseudoexfoliation Syndrome in Patients Scheduled for Cataract Surgery in Eye Camps in Yemen

**DOI:** 10.4103/0974-9233.61221

**Published:** 2010

**Authors:** Mutahar Al-Shaer, Mahfouth Bamashmus, Abdulmoghni Al-Barrag

**Affiliations:** Department of Ophthalmology, Faculty of Medicine and Health Sciences, Sana'a University, Yemen

**Keywords:** Cataract, Eye Camps, Prevalence, Pseudoexfoliation Syndrome, Yemen

## Abstract

**Purpose:**

To study the point prevalence of pseudoexfoliation syndrome (PXS) among Yemeni patients in different governorates with age-related cataract scheduled for surgery.

**Settings:**

Eye camps organized by the Nibras Health Society to perform cataract surgeries during the years 2002-2006. All patients aged 40 years and above were included in the study.

**Materials and Methods:**

A total of 2535 eyes of 2535 patients from 13 governorates, scheduled for cataract surgery in eye camps, were included. All eyes underwent complete eye examination before the surgery and were evaluated for the signs of pseudoexfoliation material in the pupil, iris and lens capsule on dilated slit lamp examination.

**Results:**

The study found 495 of the 2535 eyes (19.53%) with PXS with males more commonly affected than females (55.2 and 44.8%, respectively). The mean age of patients with PXS was 66.2 years while it was 64.6 years in non-PXS patients. The prevalence of pseudoexfoliation syndrome increased with age (10.1% in the age group of 41-50 years that increased to 28.8% in the age group of more than 81 years old). The rate of PXS detection in camps in 13 governorates ranged from 13.33 to 24.22% with an overall rate of 19.53%. The lowest rate was noticed in Sana'a and the highest in Al-Dhale governorate.

**Conclusion:**

This pilot study confirms that PXS was common in patients undergoing cataract surgery in Yemen with an increased detection rate with age. This study also highlights the prevalence of an ocular disease that is associated with systemic and ocular complications; however, further studies based on population studies are needed.

## INTRODUCTION

Pseudoexfoliation syndrome (PXS) was first described by Lindberg in 1917 in a Finnish population.^1^ The clinical diagnosis can be made by the presence of typical pseudoexfoliation material (PXM) on the anterior capsule surface of the lens and/or pupil margin. F**e**atures other than PXM include endothelial pigmentation, loss of pupillary ruff, iris sphincter transillumination, Sampaolesi’ line, and pigment deposition in the trabecular meshwork.^2^

PXS is associated with various ocular complications. Elevated intraocular pressure and glaucomatous nerve damage had been demonstrated in patients with PXS.^3^ Cataracts were reported to be more common in patients with PXS.^4^ Unfavorable factors such as poor mydriasis, zonular weakness, corneal endotheliopathy, higher rate of vitreous loss, capsular phimosis, and opacification have all been reported after cataract surgery.^5^–^9^

PXS has been the subject of quite controversy regarding its prevalence among the different ethnic groups. This ambiguity, as to the exact prevalence of PXS, seems to be due to the difference in the methods of study or survey and in the criteria for diagnosing the condition.^10^

 Clinical data, on hospital-based studies in Yemen, on PXS in patients aged 40 years above showed prevalence of 34.7^11^ and 18%.^12^ Also, there is sparse data on the prevalence of PXS in the Eastern Mediterranean Region.^13^

This is a retrospective study that examined the point prevalence of PXS in patients scheduled for cataract surgery in eye camps in Yemen. Patients with cataract only were chosen as the main target of the camp for the treatment of blinding cataract. Glaucoma cases were not included in the study since they are not treated in eye camps and are usually referred to eye centers and hospitals. The study was approved by the Ethics Committee of Nibras Health Society.

## MATERIALS AND METHODS

### Settings

All eye camps organized by Nibras Health Society during the years 2002-2006 were included in the study. A total of 2535 eyes of 2535 patients from 13 governorates who were scheduled for cataract surgery in these eye camps were included.

Inclusion criteria included patients that were above the age of 40 years with age-related cataract and scheduled for cataract surgery without prior history of intraocular surgery in the examined eye. Cataract cases secondary to systemic diseases, uveitis and trauma were excluded.

All eyes were examined by a slit lamp biomicroscope (Nidek Co. Japan) before and after mydriasis using 1.0% tropicamide eye drops (Mydriacyl, Alcon Labs, USA). The presence of PXS was confirmed by looking for the typical fluffy, white granular material at the pupillary margin, anterior capsular lens surface and surface of the iris.

For descriptive analysis, we used Statistical Package for Social Studies (SPSS 11.5, Chicago, IL). Percentage and their upper and lower confidence intervals were calculated. There was no comment recorded on the fellow eye. Patients with diabetes, traumatic cataract and less than 40 years were excluded.

## RESULTS

The mean age of patients with PXS was found to be 66.2 years and 64.6 years in the non-PXS patients. The prevalence of pseudoexfoliation syndrome increased with age. The study demonstrated that the rate was 10.1% in the age group 41-50 years, increasing to 17.9% in the age group 51-60 years, 19.0% in the age group 61-70 years, 27.2% in the age group 71-80 years and 28.8% in the age group >81 years old [Tables 1 and 2].

**Table 1:** Age distribution of studied population

Age range (years)	No. of subjects	Percentage
41-50	367	14.5
51-60	693	27.3
61-70	865	34.1
71-80	419	16.5
81 and above	191	7.6
Total	2535	100

**Table 2:** Prevalence of pseudoexfoliation stratified according to age groups

Age range (years)	Number	Percentage	Prevalence (%)
41-50	37	7.5	10.1
51-60	124	25.1	17.9
61-70	165	33.3	19.0
71-80	114	23.0	27.2
81 and above	55	11.1	28.8
Total	495	100	19.5

There were 495 eyes out of 2535 (19.53%) with pseudoexfoliation syndrome (PXS) in the patients examined in these eye camps.

Table 3 shows that males (273; 55.2%) were affected more than females (222; 44.8%). Among the 13 governorates, the prevalence of pseudoexfoliation ranged from 13.33 to 24.22%.

**Table 3:** Percentage of pseudoexfoliation patients in eye camps in different yemeni governorates

Eye camp	PXS	%(+ve)*	Percentage of total sample**	Non-PXS	%(−ve)*	Percentage of total sample**	Total sample	Percentage of total sample
Sana'a City	6	13.33	1.21	39	86.67	1.91	45	1.78
Hodeidah	42	14.24	8.48	253	85.76	12.40	295	11.64
Sana'a	19	15.97	3.84	100	84.03	4.90	119	4.69
Hajja	11	16.67	2.22	55	83.33	2.70	66	2.60
Hadramout	46	17.62	9.29	215	82.38	10.54	261	10.30
Taiz	43	17.77	8.69	199	82.23	9.75	242	9.55
Amran	36	16.90	7.27	177	83.10	8.68	213	8.40
Mahara	9	18.75	1.82	39	81.25	1.91	48	1.89
Ibb	12	20.34	2.42	47	79.66	2.30	59	2.33
Lahj	120	22.10	24.24	423	77.90	20.74	543	21.42
Mahweet	44	22.34	8.89	153	77.66	7.50	197	7.77
Dhamar	22	22.92	4.44	74	77.08	3.63	96	3.79
Dhale	85	24.22	17.17	266	75.78	13.04	351	13.85
Total	495	19.53	100.00	2040	80.47	100.00	2535	100.00

* Percentage is out of total row;

** Percentage is out of total column

The overall prevalence rate was 19.53%.The lowest (13.33%) pseudoexfoliation syndrome was noticed in Sana'a (capital of Yemen) and the highest (24.22%) prevalence was noticed in Dhale governorate.

Table 4 shows the prevalence of pseudoexfoliation syndrome with confidence intervals. Table 5 shows the prevalence of pseudoexfoliation syndrome in patients scheduled for cataract surgery in different countries.

**Table 4:** Prevalence of pseudoexfoliation syndrome with confidence intervals

Site	PXS	Prevalence	Confidence interval	
Eye camp		%(+ve)	Lower CI	Upper CI
Sana'a City	6	13.33	13.23	13.43
Hodeidah	42	14.24	14.20	14.28
Sana'a	19	15.97	15.90	16.03
Hajja	11	16.67	16.58	16.76
Hadramout	46	17.62	17.58	17.67
Taiz	43	17.77	17.72	17.82
Amran	36	16.90	16.85	16.95
Mahara	9	18.75	18.64	18.86
Ibb	12	20.34	20.24	20.44
Lahj	120	22.10	22.06	22.13
Mahweet	44	22.34	22.28	22.39
Dhamar	22	22.92	22.83	23.00
Dhale	85	24.22	24.17	24.26
Total	495	19.53	-	-

**Table 5:** Prevalence of pseudoexfoliation syndrome in patients scheduled for cataract surgery in different countries

Location	Age (years)	Number of eyes	Prevalence of pxs (%)	Reference
South Africa	>30	625	5.2	15
Turkey	>50	1480	16.4	19
Yemen (Eye camps)	>40	2535	19.53	This study
Greece	>40	509	27.9	17
Finland	>40	351	30.8	7
Yemen (Bamashmus)	>40	782	33.9	20
Estonia	>50	305	35.4	9
Ethiopia	>40	229	39.3	16

## DISCUSSION

Our study reports on the point prevalence of pseudoexfoliation syndrome (PXS) in a large chort of patients in Yemen. Although epidemiological data on the prevalence of PXS are best obtained from population-based studies, useful information on the prevalence of PXS can be obtained from different subgroups of populations, such as patients with cataract and patients with glaucoma.^9^ Camp-based studies are easy to perform as the data can be collected on a large number of patients within few days while hospital-based studies takes a long time to collect the same data and number of cases. Also camp-based studies can give an over view of different governorates visited and the population problems present while hospital-based studies are normally done on a selected group of patients mostly from one governorate or people moving to a different governorate. Such data can be used to design appropriate population-based studies.

The variation in the point prevalence of PXS among cataract to us patients attending eye camps between different governorates could be attributed to genetic,^10^,^13^ environmental^14^ and/ or geographical differences.^13^,^15^ Dhale governorate had the highest prevalence and Sana'a had the lowest and this difference could be partially related to climatic differences as Dhale governorate is considered to be one of the hottest areas in Yemen while Sana'a is considered to be one of the coolest areas in Yemen.

The prevalence rate of pseudoexfoliation in this study population was 19.53% [Table 2] which is equal to, lower, or higher than many reported studies. These variations could be explained by differences in the techniques of assessment, study of design, sampling techniques, population size, etc. It is likely that the prevalence of PXS and PXS suspects would be greater if population-based studies were performed. Globally, the reported prevalence rate of pseudoexfoliation syndrome in patients scheduled for cataract surgery [Table 5] shows extensive variations, Ethiopia (39.3%),^16^ Estonia (35.4%),^9^ Finland (30.8%),^7^ Greece (28%),^17^ South Africa (26%),^15^ Portugal (25.3%),^5^ Turkey (16.4%),^18^ and Yemen (33.9%).^19^ This reflects the variations could be due to racial, genetic, and/or geographical differences which is the likely the case in our study.

The heterogeneity in the prevalence of pseudoexfoliation among different regions of an ethnically homogeneous country has also been reported.^20^ As in our study which is supported by the hypothesis of environmental factors such as exposure to ultraviolet light and dietary factors play an important role in the pathogenesis of PXS than hereditary.^14^

PXS was more common in males (263) than in females (213). This study may be considered as different from other studies as the other reports did not mention any gender difference.^2^,^18^ It is possible that access to care which is easier for men in rural Yemen may have influenced the results of this study and a larger population study may provide information on gender differences.

The age of the patients with PXS in this study was noted to be 40 years and above and increased with age. These results agree with previous reports that suggest that the prevalence of pseudoexfoliation syndrome increases with advancing age.^17^ This does not suggest that pseudoexfoliation will not occur at younger ages. There are many studies reporting pseudoexfoliation at relatively younger age.^16^

One limitation of this study is that the rate of glaucoma in this cohort with PXS was not studied. Such studies are planned in the future.

## CONCLUSION

The prevalence rate of pseudoexfoliation syndrome among Yemeni patients with age-related cataract attending free eye camps was 19.53%, which confirms that PXS is common in Yemen but varied from one governorate to another. Population-based study should be performed to plan and tackle its complications, namely, cataract and glaucoma. Also we can consider these eye camps as a good opportunity for additional research on PXS in the future.
